# Increased expression of LncRNA BANCR and its prognostic significance in human hepatocellular carcinoma

**DOI:** 10.1186/s12957-015-0757-5

**Published:** 2016-01-12

**Authors:** Tao Zhou, Yanjing Gao

**Affiliations:** Department of Gastroenterology, Qi-Lu Hospital, Shandong University, Jinan, 250012 Shan Dong Province People’s Republic of China

**Keywords:** Long noncoding RNA, BANCR, Hepatocellular carcinoma, Prognosis

## Abstract

**Background:**

Long noncoding RNAs (lncRNAs) have been proved to play important roles in the tumorigenesis and development of human hepatocellular carcinoma (HCC). The aim of our study is to investigate the expression and function of BRAF-activated noncoding RNA (BANCR) in HCC.

**Methods:**

BANCR expression was detected in HCC tissues and cell lines by using quantitative real-time PCR (qRT-PCR). Association between BANCR levels and clinicopathological factors and patient prognosis was also analyzed. 3-(4,5-Dimethylthiazol-2-yl)-2,5-diphenyl tetrazolium bromide (MTT), flow cytometry, and transwell invasion and migration assays were used to investigate the role of BANCR in the regulation of biological behaviors of HCC cells.

**Results:**

BANCR expression was remarkably increased in HCC tissues compared with adjacent noncancerous tissues (*P* < 0.001). BANCR expression in four HCC cell lines was also significantly upregulated (*P* < 0.05). Clinicopathologic analysis revealed that high BANCR expression correlated with high tumor grade, large tumor size, venous infiltration, advanced tumor, node, and metastasis (TNM) stage, and shorter overall survival. Multivariate regression analysis identified BANCR overexpression as an independent unfavorable prognostic factor (relative risk [RR] 4.245; *P* = 0.015) in HCC patients. Moreover, BANCR downregulation in Hep3B cells impaired cell proliferation, promoted cell apoptosis, reduced cell invasion and migration, led to downregulated vimentin, and upregulated E-cadherin protein levels.

**Conclusions:**

These findings suggested that BANCR may contribute to HCC initiation and progression and would be used as not only a novel prognostic marker but also a potential therapeutic target for this disease.

## Background

Hepatocellular carcinoma (HCC) is the fifth most common cancer worldwide, with a constantly increasing frequency, especially in China [1]. Despite recent advances in clinical and experimental oncology, the long-term prognosis of HCC patients remains poor, due to late detection of disease, frequent cancer metastasis, high recurrence rate, and lack of effective therapeutic intervention for terminally staged tumors. Previous studies have revealed many HCC-associated deregulated genes and signaling pathways [2, 3], but the highly complex molecular mechanisms underlying its carcinogenesis and progression are still obscure. Therefore, it is urgent to identify reliable biomarkers of HCC for its early diagnosis, effective therapy, and prognosis evaluation.

Long noncoding RNA (lncRNA), >200 nucleotides in length, is a type of noncoding RNA molecule that can regulate gene expression in transcriptional or posttranscriptional level [4, 5]. Recent research has shown that lncRNAs participate in a large number of cellular processes, such as cell proliferation, differentiation, apoptosis, and cell cycle progression [6]. Emerging evidence indicates that lncRNAs play important roles in the biology of human cancers, which may provide a new but promising way to deal with cancer [7]. Functional lncRNAs may be applied for cancer diagnosis and prognosis and also act as potential novel therapeutic targets. For example, increased expression of lncRNA HOTTIP enhances tumor growth and migration in pancreatic cancer [8]. LncRNA MALAT1 overexpression is a negative prognostic factor for lung cancer [9]. LincRNAs VLDLR, PVT1, and GAS5 could regulate tumor cell responses to chemotherapy [10–12]. However, the understanding of the expression and function of lncRNAs in HCC is still in the early stage.

BRAF-activated noncoding RNA (BANCR), a 693-bp lncRNA, was originally identified in melanoma cells by Flockhart RJ et al. [13]. Subsequently, aberrant lncRNA BANCR expression has been confirmed in papillary thyroid carcinoma [14], retinoblastoma [15], lung cancer [16, 17], gastric cancer [18], and colorectal cancer [19]. In these tumors, BANCR regulated cell proliferation, migration, and invasion and may serve as a potential oncogene or a candidate tumor suppressor. However, no report of BANCR in HCC has been found. In the present study, we examined BANCR expression in HCC tissues and cell lines. We also investigated the correlation between BANCR levels and clinicopathological characteristics and patient’s survival. Moreover, we explored the role of BANCR in the regulation of biological behaviors of HCC cells.

## Methods

### Patients and clinical specimens

This study was approved by the Research Ethics Committee of Qi-Lu Hospital. Written informed consent was obtained from all of the patients. All specimens were handled and made anonymous according to the ethical and legal standards.

Matched fresh specimens of HCC and adjacent noncancerous liver tissues were obtained from 109 patients who underwent hepatic resection at Qi-Lu Hospital between January 2008 and March 2010. All tissues were immediately frozen in liquid nitrogen and stored at −80 °C until analysis. None of the patients had undergone chemotherapy or radiotherapy before surgery. Details of clinical and pathological characteristics of the patients are summarized in Table 1. Follow-up data were available for all patients. Overall survival was defined as the amount of time from the day of primary surgery to the date of death or the end of follow-up (for living patients).

**Table 1:** Correlation between BANCR expression and different clinicopathological features in patients with hepatocellular carcinoma

Clinicopathological features	No. of cases	BANCR expression		*P*
		Low (*n*, %)	High (*n*, %)	
Age				
<60	53	29 (54.7 %)	24 (45.3 %)	0.845
≥60	56	26 (46.4 %)	30 (53.6 %)	
Gender				
Male	80	39 (48.8 %)	41 (51.2 %)	0.666
Female	29	16 (55.2 %)	13 (44.8 %)	
Tumor grade				
G1	34	24 (70.6 %)	10 (29.4 %)	0.007
G2 + G3	75	31 (41.3 %)	44 (58.7 %)	
AFP (ng/L)				
≥400	61	27 (44.4 %)	34 (55.6 %)	0.178
<400	48	28 (58.3 %)	20 (41.7 %)	
With cirrhosis				
Yes	74	36 (48.6 %)	38 (51.4 %)	0.683
No	35	19 (54.3 %)	16 (45.7 %)	
Tumor diameter (cm)				
<5	66	41 (62.1 %)	25 (37.9 %)	0.003
≥5	43	14 (32.6 %)	29 (67.4 %)	
Tumor nodes				
Multi	37	15 (40.5 %)	22 (59.5 %)	0.16
Single	72	40 (55.6 %)	32 (44.4 %)	
Venous infiltration				
Presence	39	11 (28.2 %)	28 (71.8 %)	0.001
Absence	70	44 (62.9 %)	26 (37.1 %)	
TNM stage				
I + II	51	34 (66.7 %)	17 (33.3 %)	0.002
III	58	21 (36.2 %)	37 (66.8 %)	

### Cell culture and RNA interference

Human HCC cell lines (HuH-7, Hep3B, HepG2, and H2-M) and human normal hepatocyte CL-48 were obtained from the Institute of Biochemistry and Cell Biology of the Chinese Academy of Sciences (Shanghai, China). The cells were maintained in high glucose (4.5 g/l) Dulbecco’s modified Eagle’s medium (DMEM; Gibco-BRL, Gaithersburg, MD) supplemented with 10 % heat-inactivated fetal bovine serum (FBS), 100 U/ml of penicillin and 100 μg/ml streptomycin sulfate. Cultures were incubated in a humidified atmosphere of 5 % CO_2_ at 37 °C.

lncRNA BANCR small interfering RNA (si-BANCR) and nontargeting small interfering RNA (siRNA) (si-NC) were purchased from Sigma-Aldrich. HCC cells were transfected with siRNA by using Lipofectamine 2000 (Invitrogen, CA, USA) according to the manufacturer’s instructions. The cells were harvested for further assays 48 h after transfection.

### RNA extraction, reverse transcription, and qRT-PCR

Total RNA was extracted using the Trizol reagent (Invitrogen, Carlsbad, CA) according to the manufacturer’s instructions. RNA was reverse transcribed into cDNA using the Prime-Script one step RT-PCR kit (Takara, Dalian, China). BANCR expression levels were measured with quantitative real-time PCR (qRT-PCR) using an ABI7500 system and the SYBR Green PCR Master Mix (Takara). Glyceraldehyde-3-phosphate dehydrogenase (GAPDH) was used as an internal control. The primer sequences for BANCR were 5′-ACAGGACTCCATGGCAAACG-3′ (forward) and 5′-ATGAAGAAAGCCTGGTGCAGT-3′ (reverse). Each assay was performed in triplicate, and relative BANCR expression was normalized to GAPDH using the 2^−ΔCt^ method.

### Cell proliferation assay

Cell proliferation was analyzed using MTT assay. Briefly, approximately 1 × 10^3^ cells were seeded into a 96-well plate and incubated for 1, 2, 3, and 4 days. At the indicated time point, 20 μl of MTT (5 mg/ml) (Sigma, USA) was added into each well and incubated for another 4 h. Then, the supernatants were removed, and 150 μl of DMSO (Sigma, USA) was added to terminate the reaction. The absorbance value (OD) was measured at 490 nm on a microplate reader (Molecular Devices, Sunnyvale, CA, USA).

### Detection of apoptosis by flow cytometry

Forty-eight hours after transfection, the HCC cells were harvested, washed, and resuspended in ice-cold PBS. The cells were then treated with propidium iodide (10 μg/ml; Sigma) and Annexin V-FITC (50 μg/ml, BD) in the dark for 15 min at room temperature and examined by flow cytometry (FACScan; BD Biosciences).

### Cell invasion and migration assays

Six-well transwell chambers (8-μm pore size, Corning, New York, USA) were used to investigate cell invasion and migration. For migration assay, about 1 × 10^5^ HCC cells in serum-free media were seeded into the upper chambers after siRNA transfection. The lower chamber contained medium with 20 % FBS to stimulate migration. Following a 48 h-incubation, the cells located on the lower surface of the chamber were fixed with 95 % ethanol for 10 min, stained with 0.1 % crystal violet for 20 min, and counted using a microscope (Olympus Corp., Tokyo, Japan). For invasion assay, the upper chambers were first covered with 5 mg/ml Matrigel, and the other steps were the same as migration.

### Western blot

Cells were lysed in RIPA buffer with protease inhibitors and phosphatase inhibitors. The protein extracts were loaded onto a 10 % sodium SDS-PAGE gel and transferred to a PVDF membrane. The blots were probed with primary antibodies (Cell signal technology, USA) followed by the HRP-conjugated secondary antibody. Following three Tris-buffered saline containing 0.1 % Tween-20 (TBST) washes, the membranes were developed using ECL Plus (Millipore, MA, USA) and exposed to X-ray film. GAPDH served as the loading control.

### Statistics

All statistical analyses were performed using the SPSS 16.0 software package (SPSS, Chicago, IL, USA). The significance of differences between groups was estimated by Student’s *t* test and chi-square test. Survival curves were constructed with the Kaplan–Meier method and compared by the log-rank test. The significance of survival variables was evaluated using a multivariate Cox proportional hazards regression analysis. *P* < 0.05 was considered statistically significant.

## Results

### Increased BANCR expression in HCC tissues and cell lines

**Fig. 1:**
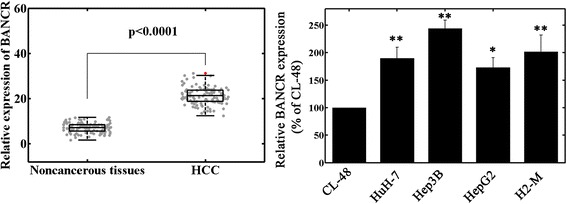
Relative BANCR expression levels in hepatocellular carcinoma (HCC) tissues and cell lines. BANCR expression was calculated by the 2^−ΔCt^ method and normalized to GAPDH. **a** BANCR expression was significantly higher in HCC tissues than in the corresponding noncancerous tissues. **b** BANCR expression was upregulated in human HCC cell lines compared to normal hepatocyte CL-48O cells. *P < 0.05; **P< 0.01

BANCR expression in HCC tissues and cell lines was measured by qRT-PCR. Figure 1a showed a significant high expression of BANCR in HCC tissues compared with adjacent noncancerous tissues (*P* < 0.001). BANCR expression in four HCC cell lines was also clearly upregulated (Fig 1b, *P* < 0.05). The Hep3B cell line, which possessed the highest BANCR expression among all tested cell lines, was chosen for the subsequent in vitro experiments.

### Association between clinicopathological characteristics and BANCR expression in HCC patients

We further analyzed the association between BANCR expression and clinicopathological characteristics of HCC. HCC samples were classified into low BANCR expression group (*n* = 55) and the high BANCR expression group (*n* = 54) according to the median BANCR expression level of all HCC samples. The association between clinicopathological characteristics and BANCR expression was summarized in Table 1. We found that high BANCR expression levels were closely correlated with high tumor grade (*P* = 0.007), large tumor size (*P* = 0.003), venous infiltration (*P* = 0.001), and advanced tumor, node, and metastasis (TNM) stage (*P* = 0.002). No significant difference was observed between BANCR expression and other clinical features such as patients’ age, gender, cirrhosis, serum AFP level, and number of tumor nodules.

### Prognostic values of BANCR expression in HCC patients

**Fig. 2:**
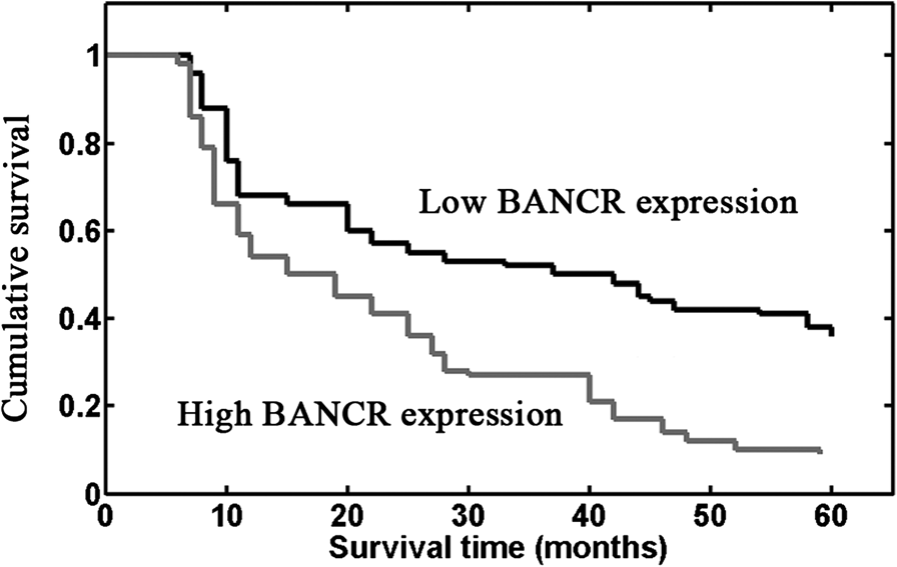
Kaplan–Meier curves for overall survival of 109 hepatocellular carcinoma patients, divided according to BANCR expression levels. High BANCR expression was significantly associated with poor survival (*P* < 0.001, log-rank test)

Then, we evaluated whether BANCR expression had prognostic potential for overall survival of HCC patients. Using the Kaplan–Meier method and log-rank test, the overall survival of patients with high BANCR expression was significantly shorter than those with low BANCR expression (*P* < 0.001; Fig. 2). Besides, the survival benefits were also found in those with small tumor diameter (*P* = 0.025), well tumor differentiation (*P* = 0.037), negative venous invasion (*P* = 0.008), and early TNM stage (*P* < 0.001; Table 2).

**Table 2:** Univariate and multivariate analysis of overall survival in 109 patients with hepatocellular carcinoma

Variables	Univariate analysis		Multivariate analysis	
	RR	*P* value	RR	*P* value
Age (years) (≥60/<60)	0.785	0.323	–	–
Gender (male/female)	0.721	0.455	–	–
Tumor grade (G1/G2 + G3)	2.387	0.037	0.795	0.146
AFP (ng/l) (≥400/<400)	0.816	0.194	–	–
With cirrhosis (yes/no)	0.904	0.092	–	–
Tumor diameter (cm) (≥5/<5)	3.252	0.025	2.655	0.039
Tumor nodes (multi/single)	0.868	0.156	–	–
Venous infiltration (presence/absence)	5.153	0.008	3.278	0.022
TNM stage (I–II/III)	6.225	<0.001	6.379	0.006
Expression of BANCR (low/high)	5.983	<0.001	4.245	0.015

Multivariate Cox regression analysis enrolling the abovementioned significant parameters revealed that BANCR expression (relative risk [RR] 4.245; *P* = 0.015), tumor size (RR 2.655; *P* = 0.039), venous infiltration (RR 3.278; *P* = 0.022), and TNM stage (RR 6.379; *P* = 0.006) were independent prognostic markers for overall survival of HCC patients (Table 2).

### Effects of BANCR downregulation on the biological behaviors of Hep3B cells

**Fig. 3:**
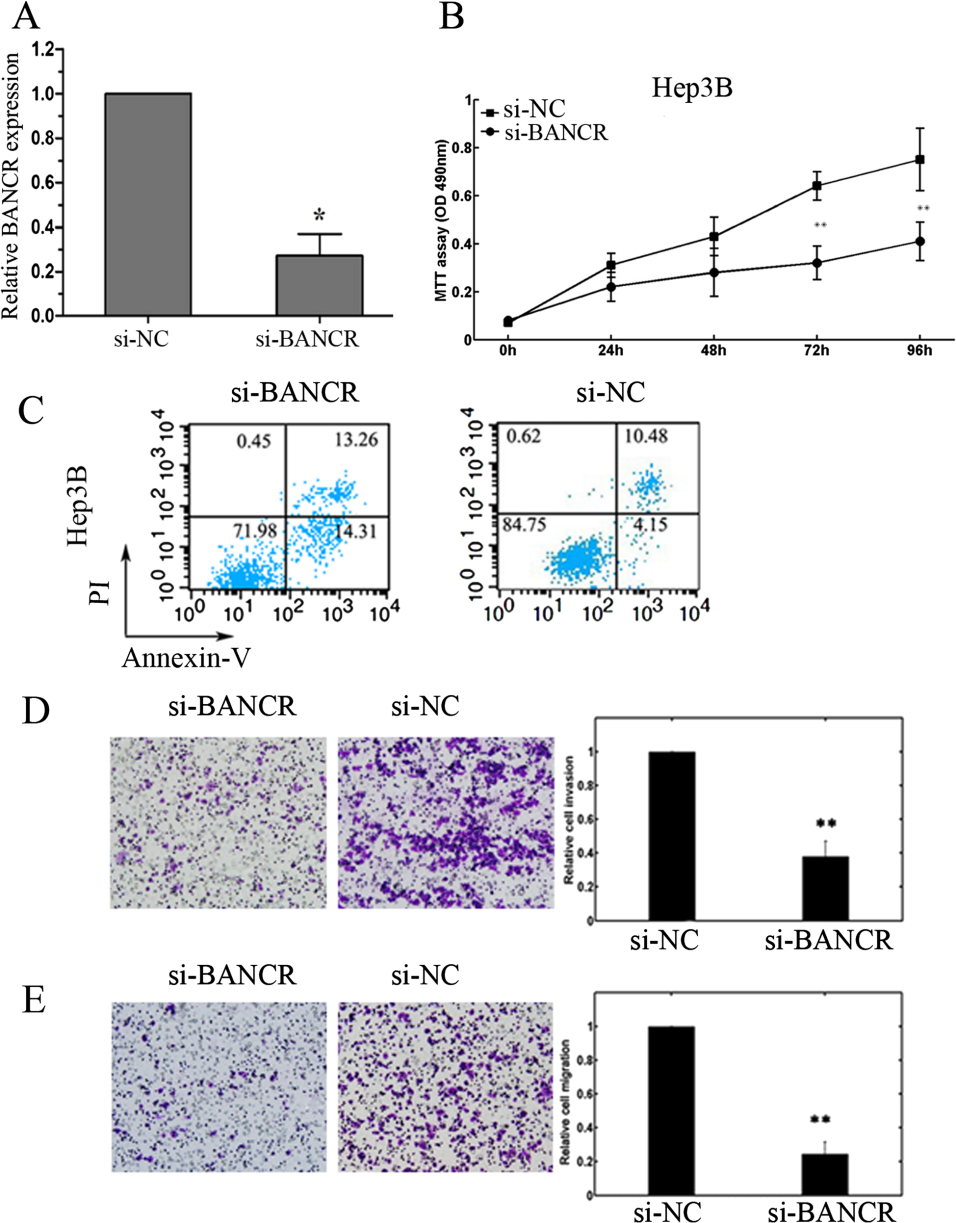
Effects of BANCR on the biological behaviors of Hep3B cells. **a** qRT-PCR analysis confirmed decreased BANCR expression in Hep3B cells after si-BANCR transfecting. BANCR expression was normalized to GAPDH. *P < 0.05. **b** Cell proliferation was measured by MTT assay in Hep3B cells transfected with si-BANCR or si-NC. **P < 0.01. **c** Flow cytometric analysis showed induced cell apoptosis after si-BANCR transfection. **d**, **e** Transwell invasion and migration assays showed that the number of invaded/migrated cells was significantly lower in the si-BANCR-transfected group than in the si-NC-transfected group. **P < 0.01

At last, we explored the role of BANCR in regulating the biological behaviors of HCC cells. BANCR expression in Hep3B cells was evidently downregulated after si-BANCR transfection (Fig 3a). As shown in Fig. 3b, c, BANCR downregulation impaired Hep3B cell proliferation and promoted cell apoptosis compared to the si-NC group. In addition, the invasion and migration ability of Hep3B cells was significantly reduced after si-BANCR transfection (Fig 3d, e).

### BANCR induces Epithelial-to-mesenchymal transition (EMT) phenotypes

**Fig. 4:**
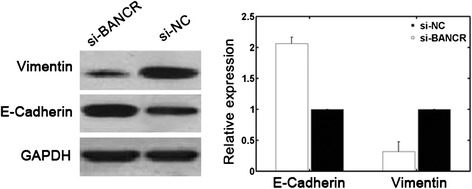
BANCR downregulation in Hep3B cells was associated with decreased vimentin and increased E-cadherin protein levels

To explore the potential underlying mechanisms of BANCR-related high cell migration, we detected the changes in E-cadherin and vimentin protein levels after si-BANCR transfection by Western blot. The results showed that BANCR downregulation in Hep3B cells was associated with upregulated E-cadherin and downregulated vimentin protein levels (Fig. 4).

## Discussion

Identifying novel molecules that take part in HCC formation and progression may be helpful for improving the diagnosis, prevention, and treatment of this disease. The relationship between lncRNAs and tumors has currently become one of the focuses of cancer studies. Abnormal expression of several lncRNAs has been reported in HCC. For example, lncRNA AOC4P overexpression in HCC cells significantly reduced cell proliferation, migration, and invasion by inhibiting the epithelial-mesenchymal transition (EMT) [20]. lncRNA UCA1 was aberrantly upregulated in HCC tissues and associated with TNM stage, metastasis, and postoperative survival [21]. UCA1 depletion inhibited the growth and metastasis of HCC cell lines in vitro and in vivo. Serum lncRNA-AF085935 was helpful to discriminate HCC from hepatitis B viral infected patients and healthy controls [22]. Injection of the lncRNA PTENP1-expressing vectors into mice bearing HCC tumors effectively mitigated the tumor growth, suppressed intratumoral cell proliferation, elicited apoptosis, autophagy and inhibited angiogenesis [23]. These findings suggested that lncRNAs might play important roles in HCC initiation and development and have a great potential for clinical application.

In the present study, we observed a high BANCR expression in HCC specimens and cell lines, providing the first evidence that BANCR overexpression was closely associated with HCC carcinogenesis. Then, we correlated increased BANCR levels with aggressive clinicopathological features of HCC tissues. Downregulation of BANCR in Hep3B cells would reduce cell proliferation, enhance cell apoptosis, and impair cell invasion and migration. These findings revealed that BANCR might be involved in HCC progression and contribute to molecular-targeted therapy. In addition, our research showed that HCC patients with high BANCR levels tended to have shorter overall survival than patients with lower levels. Multivariate Cox hazard regression analysis identified high BANCR expression as an independent indicator of unfavorable prognosis. To our knowledge, this is the first study to analyze the expression and clinical significance of BANCR in HCC.

BANCR has been reported as an oncogene in several types of human malignancies. BANCR levels in human malignant melanoma tissues increased with advanced tumor stages, and the knockdown of BANCR suppressed melanoma cell proliferation and migration through MAPK pathway [13, 24]. BANCR overexpression correlated with tumor stage and lymph node metastasis in colorectal cancer and contributed to cancer cell migration through inducing epithelial-mesenchymal transition. In gastric cancer, BANCR expression was increased in tumor tissues compared with paired adjacent normal tissues. High BANCR levels were positively associated with clinical stage, tumor depth, lymph node and distant metastasis, and poor prognosis [18]. In retinoblastoma, BANCR regulated cell proliferation, migration, and invasion in vitro and overexpressed BANCR expression linked with tumor size, choroidal invasion, and optic nerve invasion [15].

In contrast to the tumor-promoting properties mentioned above, Sun et al. reported that BANCR was obviously downregulated in non-small cell lung cancer tissues and that reduced BANCR expression was associated with larger tumor size, lymph node metastasis, advanced TNM stage, and shorter overall survival. Ectopic expression of BANCR impaired cell viability and invasion, leading to the inhibition of metastasis in vitro and in vivo [16]. Taken together, the role of lncRNA BANCR in human malignancies may be multifaceted and determined by the distinct context in various microenvironments.

EMT is one of the key processes for primary tumor cells to acquire a migratory capacity [25]. Reduced E-cadherin expression and increased vimentin expression are associated with HCC progression [26, 27]. Emerging evidence demonstrates that lncRNAs, including BANCR, may regulate EMT processes in several cancer types [19, 28–30]. In the present study, BANCR downregulation resulted in upregulated E-cadherin and downregulated vimentin protein levels. These findings indicated that BANCR might be involved in the regulation of EMT in HCC, providing a possible explanation for BANCR-related high cell migration.

## Conclusions

In conclusion, our research confirmed increased BANCR expression in HCC tissues and cell lines. Our study also showed that high BANCR levels correlated tumor progression and poor prognosis. Regulation of BANCR expression influenced biological behaviors of HCC cells. These findings suggested that BANCR may act as an oncogene in HCC initiation and development and would be not only a novel prognostic marker but also a potential therapeutic target for this disease.
