# Expression of Concern to: Increased expression of LncRNA BANCR and its prognostic significance in human hepatocellular carcinoma

**DOI:** 10.1186/s12957-019-1644-2

**Published:** 2019-06-13

**Authors:** Tao Zhou, Yanjing Gao

**Affiliations:** Department of Gastroenterology, Qi-Lu Hospital, Shandong University, Jinan, Shan Dong Province 250012 People’s Republic of China

**Expression of Concern to: World J Surg Oncol**

**https://doi.org/10.1186/s12957-015-0757-5**

The Editor-in-Chief is issuing an editorial expression of concern to alert readers that the following articles published within a very close time frame contain similarities in text and figures to this article [1]. Image si-BANCR in Figs. 3D and 4 in article [2] are very similar to images presented in Fig. 3D as well as the western blot in Fig. 4 in this article. Fig. 3C is very similar to Fig. 3C in retracted article [3]. Image si-NC in Fig. 3E in article [4] is very similar to Fig. 3D of this article. Significant text overlap has been found in articles [5, 6] with this article. The authors have stated that a language editing company submitted a wrong article on their behalf. The matter has been referred to the authors’ institution for further investigation.

None of the authors agree to this EEoC.
